# As “Cinco Ondas Malignas” da Eletrocardiografia

**DOI:** 10.36660/abc.20220649

**Published:** 2023-02-23

**Authors:** Mitermayer Reis Brito

**Affiliations:** 1 Hospital Madre Teresa Belo Horizonte MG Brasil Hospital Madre Teresa - Arritmias/Eletrofisiologia, Belo Horizonte, MG – Brasil

**Keywords:** Diagnóstico por Imagem/métodos, Eletrocardiografia/métodos, Arritmias Cardíacas, Taquicardia Ventricular, Síncope, Morte Súbita

As “Cinco Ondas Malignas da Eletrocardiografia” são na sua maioria causadas por um substrato genético não bem conhecido, que são definidas como um novo subgrupo de condições cardíacas, classificadas como doenças arritmogênicas hereditárias.^1 , 2^

Algumas das síndromes arritmogênicas hereditárias apresentam uma onda clássica no eletrocardiograma (ECG), as quais podem ser uma das principais anormalidades eletrocardiográficas para o seu diagnóstico.^1 , 3 - 12^

A intenção deste ponto de vista é ressaltar a importância de agrupar estas ondas em um conceito ainda não descrito, e mais didático, demonstrando as alterações eletrocardiográficas presentes nestas doenças, que ocorrem no complexo QRS, segmento ST e onda T. Seria como surfar nestas ondas ( Figura1 -Ilustrativa) e assim facilitar a identificação das suas principais características eletrocardiográficas em derivações específicas no plano frontal e horizontal do ECG.

**Figura 1:**
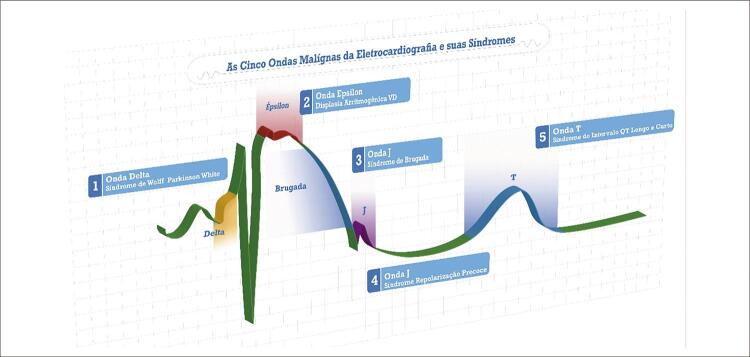
– Cinco Ondas Malignas da Eletrocardiografia.

As manifestações eletrocardiográficas das síndromes relacionadas às Cinco Ondas Malignas podem ocorrer em vários fenótipos e apresentarem um caráter lento e progressivo de suas evoluções clínicas, assim como suas respectivas alterações eletrocardiográficas, dificultando suas identificações e diferenciações com outros fenótipos não cardíacos (ex: miopatia esquelética, outras patologias sistêmicas) cujas doenças subjacentes podem ter causas genéticas (ex: miopatia gen desmin) e não genéticas (ex: Doença Chagas).^2 , 12^

As “Cinco Ondas Malignas” podem ter como principal desfecho clínico arritmias com risco de vida como taquicardia ventricular, fibrilação ventricular e manifestações clínicas como síncope e morte súbita.^1 , 3 , 4 , 10 - 13^

## Principais características ECG das cinco ondas malignas e suas síndromes

**Figura 2:**
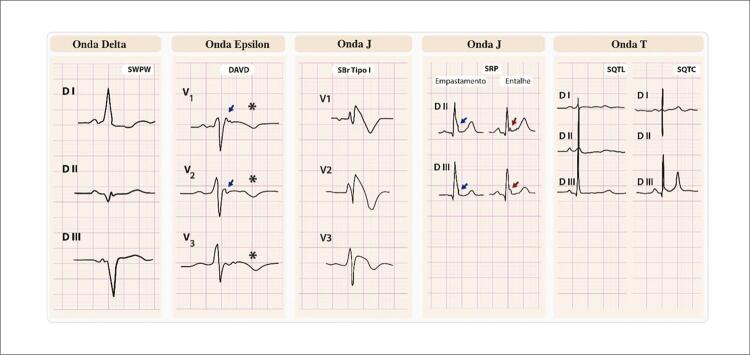
– Principais características ECG das cinco ondas malignas. SWPW: Síndrome Wolff Parkinson White; DAVD: displasia arritmogênica ventrículo direito; SBr: Síndrome Brugada; SRP: Síndrome Repolarização Precoce; SQTL: Síndrome QT Longo; SQTC: Síndrome QT Curto, *ondas T negativas, ↙onda Epsilon.

As principais características ECG das Cinco Ondas Malignas e suas síndromes estão apresentadas na Figura 2 .

### Onda Delta e a Síndrome de Wolff Parkinson White (SWPW)

As principais manifestações eletrocardiográficas da SWPW são decorrentes da pré excitação ventricular através de uma via acessória átrio ventricular e consistem no encurtamento do intervalo PR (≤0,12s), empastamento na porção inicial do QRS (onda delta), alargamento do QRS (≥0,12s) e alterações secundárias do segmento ST/T.^5^

### Onda Epsilon e a Displasia Arritmogênica do Ventrículo Direito (DAVD)

As ondas épsilon refletem o atraso de condução no VD devido à substituição do miocárdio por tecido fibrótico gorduroso e são potenciais elétricos pós-excitação ventricular de pequena amplitude em V1a V3, que ocorrem entre o final do QRS e início do segmento ST e são observados em até 25% dos casos.^6 , 12^

A despolarização é anormal e consiste na duração da ativação terminal, que é o mais longo valor nas derivações V1-V3, do nadir da onda S ao final de todas as deflecções da despolarização (≥55ms), portanto incluindo a porção ascendente da onda S, os potenciais fracionados tardios e a onda epsilon. Além da onda epsilon, pode ocorrer presença de ondas T invertidas em V1, V2 V3, acima da idade de 14 anos (com BRD incompleto) até 67% dos casos. A presença da duração de ativação terminal anormal e de ondas T invertidas são consideradas anormalidades de maior risco para arritmias.^7 , 12^

## A onda J e suas Síndromes

As síndromes da onda J incluem a Síndrome de Brugada (SBr) e a Síndrome da Repolarização Precoce (SRP) e são caracterizadas pela manifestação de uma proeminente onda J no ECG, ocorrendo como uma elevação do segmento ST e surgimento de arritmias com risco de vida.^8 - 10^ A SBr e a SRP diferem em relação a magnitude e localização das derivações da onda J anormal.^10^

Estas anormalidades eletrocardiográficas tem sido descritas por estudos como um potente gradiente da repolarização localizado sobre as regiões ínfero lateral do ventrículo esquerdo em pacientes com a SRP associados a uma ativação ventricular normal. Contrariamente, foram registrados uma atividade fragmentada do eletrograma devido à condução lenta por descontinuidade na via de saída do ventrículo direito de pacientes com a SBr somados a uma significativa dispersão de gradiente da repolarização.^8^

### Onda J e a Síndrome de Brugada

A única forma diagnóstica ECG da SBr é o Tipo I (“tipo Coved”) caracterizado pela elevação do segmento ST ≥ 2mm (0,2mV) com uma onda J larga e elevada em uma ou mais derivações precordiais direitas, posicionadas no quarto espaço intercostal (V1e/ou V2) ou em posições mais craniais (2º, 3º espaço intercostal).^9 , 10^

### Onda J e a Síndrome da Repolarização Precoce

A SRP é reconhecida por uma onda J distinta, com início elevado, entalhe ou empastamento da porção terminal do QRS e o segmento ST em 2 ou mais derivações contínuas excluindo V1-V3; o final da onda J é elevado (≥ 0,1mV) nas derivações laterais (Tipo I), infero lateral (Tipo II) ou infero lateral mais as derivações precordiais direitas (Tipo III). Pacientes com a onda J muito proeminente e o segmento ST horizontalizado ou descendente, estão associados com pior prognóstico.^8 , 12^

### Onda T e as Síndromes do QT longo/Curto

O diagnóstico da Síndrome do QT longo (SQTL) é baseado no ECG e nas medidas do intervalo QT corrigido para a frequência cardíaca (QTc). Os valores de corte têm sido estabelecidos baseados no uso da Fórmula de Bazett. Normalmente, o QTc anormal é maior ou igual a 460ms nas mulheres e 440ms nos homens. Adicionalmente, pacientes afetados pela SQTL congênita demonstram frequentes anormalidades na morfologia da onda T, como ondas T difásicas, entalhes, baixa amplitude, ou início da sua inscrição muito lento. A alternância das ondas T é raro, mas isto está correlacionado com pior prognóstico. O intervalo QT é o mais importante indicador de risco. Pacientes que apresentam com QTc > 500ms repetitivamente são considerados de alto risco para arritmias e morte súbita1. O ponto de corte de limite para definição da SQT curto tem sido considerado entre 340 a 360ms. A maioria dos pacientes apresentam ondas T apiculadas, de maior amplitude e de curta duração e praticamente uma ausência do segmento ST e o intervalo Tpeak-Tend relativamente longo.^3 , 4 , 13^

## Como explicar as arritmias cardíacas das Cinco Ondas Malignas e suas Síndromes?

As mutações genéticas dos canais iônicos, que ocorrem nas canolopatias (SBr, SQTL/Curto) e na SRP, alteram o fluxo iônico, com modificações na despolarização e repolarização ventricular, e podem levar a arritmias malignas.^1 , 3 , 4 , 10 , 12 , 13^

Os danos ao miocárdio com substituição por tecido fibroso/gorduroso na DAVD, podem induzir arritmias com risco de vida.^6 , 7^

O risco de morte súbita na SWPW é raro e deve-se à presença de fibrilação/flutter atrial pré-excitados pela via acessória com parada cardíaca como mecanismo documentado.^5^ A associação entre a SWPW e a hipertrofia ventricular tem sido descrita como uma variante genética, podendo estar associado a doenças do sistema de condução, caracterizando a Síndrome PRKAG2, com presença de taquiarritmias frequentes, morte súbita e BAV total.^11^

## Cenário das mudanças das síndromes arritmogênicas hereditárias

As síndromes arritmogênicas que ocorrem nas Cinco Ondas Malignas são hereditárias e tem sido normalmente atribuída na sua maioria, a uma herança dominante autossômica, devido a anormalidades monogenicas.

Estas doenças podem ser geneticamente mais complexas e oligogenicas, especialmente na SBr. A simples equação de genótipo é igual fenótipo/doença tem sido também progressivamente questionada. Isto foi inicialmente evidenciado pela superposição do gene SCN5A não somente à SBr, como também a doença progressiva da condução cardíaca (DPCC) e SQTL acompanhados da associação com outros fenótipos, como a miocardiopatia dilatada e a fibrilação atrial.

Os critérios para determinar a patogenecidade da maioria destas doenças têm sido bastante rigorosos, não mais considerando uma variável binaria (genótipo é igual a fenótipo), mas ao invés, um espectro amplo de patogenecidade.^14^ Isto significa que sob um gene (exemplo: SCN5A) podem estar presentes outras síndromes além da SBr, cujas manifestações clínicas poderiam ocorrer em diferentes estágios das suas evoluções devido a mutações genéticas. A implicação destes critérios na decisão clínica destas síndromes é relevante, uma vez que a prevalência destas cinco entidades clinicas é baixa como um todo, os seus diagnósticos não são geralmente simples, mas os seus desfechos clínicos podem ser fatais.

## References

[1] El Kaufman (2009). Mechanisms and clinical management of inherited channelopathies Long QT syndrome, Brugada syndrome, catecholaminergic polymorphic ventricular tachycardia, and short QT Syndrome. Heart Rhythm.

[2] Brito MR, Miranda CE, Rabelo W, Marino R (2010). Type 1 electrocardiographic Brugada pattern in a woman with Chagas disease: a case report. Europace.

[3] Cerrone M, Cummings S, Alansari T, Priori S (2012). A clinical approach to inherited arrhythmias. Circ Cardiovasc Genet.

[4] Priori S (2013). HRS/EHRA/APHRS Expert Consensus Statement on the Diagnosis and Management of Patients with Inherited Primary Arrhythmia Syndromes. Heart Rhythm.

[5] Abdelghani S, Rosenthal T, Morin D (2016). Surface Electrocardiogram Predictors of Sudden Cardiac Arrest. Ochsner J.

[6] Marcus FI, Fontaine G, Guiradon G, Frank R, Laurenceau JL, Malergue C (1982). Right ventricular dysplasia: a report of 24 adult cases. Circulation.

[7] Gandjbakhch E, Redheuil A, Pousset F, Charron P, Frank R (2018). Clinical Diagnosis, Imaging, and Genetics of Arrhythmogenic Right Ventricular Cardiomyopathy/Dysplasia: JACC State-of-the-Art Review. J Am Coll Cardiol.

[8] Antzelevitch C, Yan G, Ackerman M, Borggrefe M, Corrado D, Guo J (2017). J-Wave syndromes expert consensus conference report: Emerging concepts and gaps in knowledge. Europace.

[9] Brugada P, Brugada J (1992). Right bundle branch block, persistent ST segment elevation and sudden cardiac death: a distinct clinical and electrocardiographic syndrome: a multicenter report. J Am Coll Cardiol.

[10] Diego J, Antzelevitch C (2018). J wave syndromes as a cause of malignant cardiac arrhythmias. Pacing Clin Electrophysiol.

[11] Van der Steld L, Campuzano O, Serra A, Zamorano M, Matos S, Brugada R (2017). Wolff-Parkinson-White Syndrome with Ventricular Hypertrophy in a Brazilian Family. Am J Case Rep.

[12] Towbin J, McKenna W, Abrams D, Tintelen J, Wilde A, Zareba W (2019). HRS Expert consensus statement on evaluation, risk stratification, and management of arrhythmogenic cardiomyopathy. Heart Rhythm.

[13] Stiles M, Wilde A, Abrams D, Acker M, Albert C, Behr E (2021). 2020 APHRS/HRS expert consensus statement on the investigation of decedents with sudden unexplained death and patients with sudden cardiac arrest, and of their families. Heart Rhythm.

[14] Gray B, Behr E (2016). New Insights Into the Genetic Basis of Inherited Arrhythmia Syndromes. Circ Cardiovasc Genet.

