# Supplementary material for: As “Cinco Ondas Malignas” da Eletrocardiografia
Source: Arq Bras Cardiol. 2023 Feb 23;120(3):e20220649. [Article in Portuguese] doi: 10.36660/abc.20220649 (PMC9972877; doi:10.36660/abc.20220649)
Supplement: Translation

# Translation: The “Five Malignant Waves” of the Electrocardiography

**Authors:** Mitermayer Reis Brito

**Affiliations:** 1 Brasil Hospital Madre Teresa - Arritmias/Eletrofisiologia, Belo Horizonte, MG – Brasil

**Keywords:** Diagnostic Imaging/methods, Electrocardiography/methods, Arrhythmias, Cardiac, Tachycardia, Ventricular, Syncope, Death Sudden

The “Five Malignant Waves of Eletrocardiography **”** are in their majority due a genetic substrate not well known, which are considered as a new subgroup of cardiac conditions, classified as inherited arrhythmogenic diseases.^1 , 2^

Some of the inherited arrhythmogenic syndromes present a classical wave on the electrocardiogram (ECG), which may be one of the main ECG abnormalities for their diagnostics.^1 , 3 - 12^

The aim of this point of view is to highlight the importance to gather these waves in a new concept not described before and more didactic, presenting the ECG abnormalities which occur on QRS complex, ST segment and T wave.

It would be as to surf over these waves along QRS complex, ST segment and T wave and so facilitate the identification of their main ECG features, in specific leads in the frontal/horizontal planes.

The ECG manifestations of the syndromes related to “Five Malignant Waves “ ( Figure 1 Ilustrative) may occur in several phenotypes, present slow and progressive clinical evolutions as well their respectives ECG changes, making more difficult their identifications and diferentiations with others noncardiac phenotypes (e.g.skeletal myopathy, other organ patologies) which underlying diseases may have genetic (e.g. desmin gen myopathy) and non genetic (e.g. Chagas disease) causes.^2 , 12^

**Figure 1:** – Five Malignant Waves of Eletrocardiography.

The “Five Malignant Waves” may have as clinical outcomes life-threatening arrhythmias as polymorphic ventricular tachycardia, ventricular fibrillation and clinical manifestations as syncope and sudden death.^1 , 3 , 4 , 10 - 13^

## The main ECG features of the five malignant waves and their syndromes

The Main ECG features of the “Five Malignant Waves” and their syndromes are presented in Figure 2 .

**Figure 2:** – Main ECG features of the five malignant waves. WPWS: Wolff Parkinson White Syndrome; RVAD: Right Ventricle Arrhythmogenic Displasia; BrS: Brugada Syndrome; ERS: Early Repolarization Syndrome; LQTS: Long QT Syndrome; SQTS: Short QT Syndrome, *negative T waves, ↙Epsilon wave.

### Delta Wave and Wolff Parkinson White Syndrome (WPWS)

The main ECG features of WPWS are due to the ventricle pre excitation by the presence of an atrio-ventricular accessory pathway and consist the shortening of PRinterval(≤0,12s), slurring in the initial portion of QRS (delta wave), widening of QRS(≥0.12s) and secondary of ST/T segment changes.^5^

### Epsilon wave and the Right Ventricle Arrhythmogenic Displasia (RVAD)

The epsilon waves reflect delay conduction in RV due to replacement of myocardial by fibrotic fatty tissue and are electrical deflections pos ventricular excitation, of low amplitude in V1 to V3, which occur between the end of the QRS and the onset of ST segment and are observed up to 25% of the cases.^6 , 12^

The depolarization is abnormal and consists on duration of terminal activation, which is the longest value in leads V1-V3, from the nadir of the S wave to the end of all depolarization deflections (≥55ms), therefore including not only the ascendent portion of S wave, but as well the late fractioned potentials and the epsilon wave.

Besides the épsilon wave, may occur inverted T waves in leads V1,V2,V3, >14 years of age (with incomplete RBBB) or in V4,V5,V6 and inverted T wave in V1,V2,V3,V4 in the presence of complete RBBB up to 87% of the cases. The presence of abnormal duration of terminal activation and inverted T wave are considered abnormalities of major risk for arrhythmias.^7 , 12^

## The J wave and their Syndromes

The J wave syndromes, including the Brugada Syndrome (BrS) and the early repolarization syndrome(ERS) are caracterized by the manifestation of prominent J waves on the ECG and the development of life-threatening cardiac arrhythmias.^8 - 10^

The BrS and the ERS differ with respect to the magnitude and lead location of abnormal J waves.^10^

These ECG abnormalities have been reported by studies as very steep localized repolarization gradients across the inferior/lateral regions of LV of ERS patients, in conjuction with normal ventricular activation. In contrast, fractionated electrogram activity due to slow discontinuous conduction was recorded in the right ventricle outflow tract of BrS patients in addition to a steep dispersion of repolarization gradientes.^8^

### J wave and Brugada Syndrome

The only ECG form diagnostic of BrS is a Type 1 (“coved type”) ST segment elevation of ≥ 2mm (0.2mV) with a broad and elevated J wave, in one or + right precordial leads positioned in the 4 th intercostal space (V1 and/or V2) or in more cranial positions (2nd or 3rd intercostal space).^9 , 10^

### J Wave and the Early Repolarization Syndrome

The ERS is recognized with the appearance of distint J wave with elevated onset, notch or slur on the terminal part of QRS and the ST segment in two or more contiguous ECG leads, excluding V1-V3; the end of the J wave are elevated (>=0.1mV) in the lateral (Type 1), infero-lateral (Type II) or in infero-lateral + right precordial leads (Type III). Patients with a very prominent J wave and horizontal or descending ST segment are associated with worse prognosis.^8 , 12^

### T Wave and LQTS / SQTS

The diagnosis of LQTS is based on the ECG and the measurement of the heart rate corrected QT interval (QTc). Cut-off values have been established based on the use of Bazett formula. Normally, an abnormal QTc are greater than or equal to 460ms in females and 440ms in males. Furthermore, patients affected by congenital LQTS show frequent abnormalities in the T wave morphology, as diphasic T waves, notches, low amplitude, or very slow onset. The alternation of T wave is rare, but it correlates with poor prognosis.

The QT interval is the most important indicator of risk. Patients who present with QTc >500ms repetitively are considered at high risk for arrhythmias and SCD.

The cut-off for the definition of SQTS has been raised to values between 340 to 360ms. Most patients show tall, peaked, and narrow T waves, with an almost absent ST segment and a relative long Tpeak-Tend interval.^3 , 4 , 13^

## How to explain the Cardiac Arrhythmias of “Five Malignant Waves” and their Syndromes?

The genetics mutations of the ionic channel which are present in the canelopathies (BrS, LQTS, SQTS) and ERS lead to the ionic flow alterations, with ventricular depolarization and repolarization changes and may lead to malignant arrhythmias.^1 , 3 , 4 , 10 , 12 , 13^The injuries to the myocardial by fatty/fibrotic tissue replacement in RVARD, may induce life-threatening arrhythmias.^6 , 7^The sudden death risks in the WPWS are rare and are due to the presence of pre-excited atrial fibrillation/flutter by the accessory pathway with cardiac arrest as the documented mechanism.^5^The association between WPWS and ventricular hypertrophy has been described as a genetic variant, and may been associated to the presence of frequent tachyarrhythmias, sudden death and complete AV Block.^11^

## The Landscape of the Inherited Arrhythmogenic Syndromes Changes

The arrhythmogenic syndromes which occur in the Five Malignant Waves are inherited and are normally assigned in their majority to autosomal dominant inheretance because of monogenic abnormalities.

These diseases may be genetically more complex and oligogenic, particularly in BrS. The simple genotype equals fenotype/disease equation is also increasingly questioned. This was first evidenced by SCN5A overlap not only of BrS, as well as progressive cardiac conduction disease (PCCD), and LQTS followed by association with other fenotypes, as dilated cardiomyopathy and atrial fibrillation

The criteria to determine the pathogeneticy of the majority of these diseases have been stringent, and no longer considered as a binary variable (genotype equals fenotype), but instead a wide spectrum of pathogeneticy.^14^It means that under one gen (i.e. SC5NA) may be present others syndromes beyond BrS which the clinical manifestions may occur in different stages of their evolutions due to genes mutations. The implication of these criteria on the clinical decision of these syndromes is relevant, once the overall prevalence of these five clinical entities is low, their diagnosis are not always simple but the clinical outcomes can be fatal.
